# A novel retinoic acid receptor-γ agonist antagonizes immune checkpoint resistance in lung cancers by altering the tumor immune microenvironment

**DOI:** 10.1038/s41598-023-41690-5

**Published:** 2023-09-09

**Authors:** Cheng-Hsin Wei, Lu Huang, Blair Kreh, Xiuxia Liu, Liliya Tyutyunyk-Massey, Masanori Kawakami, Zibo Chen, Mi Shi, Serguei Kozlov, King C. Chan, Thorkell Andresson, Mary Carrington, Vidyasagar Vuligonda, Martin E. Sanders, Amir Horowitz, Patrick Hwu, Weiyi Peng, Ethan Dmitrovsky, Xi Liu

**Affiliations:** 1 Molecular Pharmacology Program, Frederick National Laboratory for Cancer Research, PO Box B, Frederick, MD 21701 USA; 2 Department of Melanoma Medical Oncology, The University of Texas MD Anderson Cancer Center, Houston, TX USA; 3 Center for Advanced Preclinical Research, Frederick, MD USA; 4 Protein Characterization Laboratory, Frederick, MD USA; 5 Basic Science Program, Frederick National Laboratory for Cancer Research, Frederick, MD USA; 6 Laboratory of Integrative Cancer Immunology, Center for Cancer Research, National Cancer Institute, Bethesda, MD USA; 7 Io Therapeutics, Inc., Spring, TX USA; 8 Icahn School of Medicine at Mount Sinai, New York, NY USA; 9 Division of Cancer Medicine, The University of Texas MD Anderson Cancer Center, Houston, TX USA; 10 Present Address: Moffitt Cancer Center, Tampa, FL USA

**Keywords:** Lung cancer, Tumour immunology, Preclinical research, Drug development

## Abstract

All-*trans*-retinoic acid (ATRA), the retinoic acid receptors (RARs) agonist, regulates cell growth, differentiation, immunity, and survival. We report that ATRA-treatment repressed cancer growth in syngeneic immunocompetent, but not immunodeficient mice. The tumor microenvironment was implicated: CD8^+^ T cell depletion antagonized ATRA’s anti-tumorigenic effects in syngeneic mice. ATRA-treatment with checkpoint blockade did not cooperatively inhibit murine lung cancer growth. To augment ATRA’s anti-tumorigenicity without promoting its pro-tumorigenic potential, an RARγ agonist (IRX4647) was used since it regulates T cell biology. Treating with IRX4647 in combination with an immune checkpoint (anti-PD-L1) inhibitor resulted in a statistically significant suppression of syngeneic 344SQ lung cancers in mice—a model known for its resistance to checkpoints and characterized by low basal T cell and PD-L1 expression. This combined treatment notably elevated CD4^+^ T-cell presence within the tumor microenvironment and increased IL-5 and IL-13 tumor levels, while simultaneously decreasing CD38 in the tumor stroma. IL-5 and/or IL-13 treatments increased CD4^+^ more than CD8^+^ T-cells in mice. IRX4647-treatment did not appreciably affect in vitro lung cancer growth, despite RARγ expression. Pharmacokinetic analysis found IRX4647 plasma half-life was 6 h in mice. Yet, RARα antagonist (IRX6696)-treatment with anti-PD-L1 did not repress syngeneic lung cancer growth. Together, these findings provide a rationale for a clinical trial investigating an RARγ agonist to augment check point blockade response in cancers.

## Introduction

All-*trans*-retinoic acid (ATRA) is the vitamin A metabolite that regulates diverse signaling pathways through binding to the nuclear retinoic acid receptor (RAR) and retinoid X receptor (RXR) complex^1^. ATRA treatment of acute promyelocytic leukemia (APL) is an example of retinoid-based differentiation therapy that became approved by the FDA, changing the natural history of this leukemia^2–7^. Depending on the ratio of cellular retinoic acid binding protein II (CRABP-II) and fatty acid binding protein 5 (FABP5) expression profile, ATRA-treatment can favor activation of the RAR or peroxisome proliferator-activated receptor-β/γ (PPARβ/γ). This causes opposing effects on tumor growth^8,9^. In cancer cells with a high ratio of CRABP-II/FABP5 expression, ATRA functions in a proapoptotic manner, eliciting anti-neoplastic activity. Conversely, cancer cell survival is enhanced when a low ratio of CRABP-II/FABP5 expression profiles exist^8,9^. Hence, cancer cells exhibit opposing fates based on the dual transcriptional activation properties of ATRA.

The immune system is regulated by ATRA-treatment^10^. Depending on the microenvironment and the specific cytokine signals, ATRA exerts distinct effects on T cell response and tumorigenicity^10^. ATRA-treatment produced by intestinal dendritic cells and macrophages can promote differentiation of immune-suppressive fork head box P3 (Foxp3) regulatory T cells from naive T cells, thereby inhibiting generation of pro-inflammatory Th1 and/or Th17 cells^10–13^.

ATRA maintains barrier integrity and mucosal tolerance in immune homeostasis. During inflammation or infection, ATRA cooperates with inflammatory cytokines to promote Th1, Th2, and Th17 cell differentiation from naive T cells, recruiting innate cell populations and antigen-presenting cells^10,12,13^. Thus, ATRA has a major role in the regulation of inflammation by altering cytokine production, modulating T helper cell responses, and protecting against infection and tissue damage.

Immune checkpoint blockade changed oncology practice through anti-tumor activity of monotherapy or combination therapy against some malignancies^14–16^. Antibodies binding programmed death-1 (PD-1) receptor on T cells or programmed death-ligand 1 (PD-L1) on tumor cells can activate immune response against cancers^14–16^. There is a need to find ways to augment these anti-tumor responses of checkpoint blockade, as studied here.

Retinoids regulate immune response, as we and others reported to occur by causing direct effects on T cells^17–21^. Selective RAR agonists or antagonists have not yet been comprehensively explored as to how these agents affect anti-tumor response to either PD-1 or PD-L1 treatment. This knowledge gap is examined here by elucidating the consequences of treatment with a novel RARγ agonist on the growth of syngeneic murine cancer models. Prior work found that RARγ is critical for T and B lymphocyte development and RARγ can alter inflammatory cytokine production^22,23^.

We report here that ATRA-treatment statistically-significantly repressed growth of murine syngeneic models of lung cancer, colon cancer and melanoma. In marked contrast, ATRA’s antineoplastic effects were not observed in immunodeficient mice or in syngeneic mice that had cytotoxic T cell depletion. These findings implicated changes in the tumor immune microenvironment in engaging these anti-neoplastic effects. Notably, combining ATRA-treatment with anti-PD-1 blockade did not statistically-significantly cooperate in repressing tumor growth in mice bearing syngeneic cancers. We sought to augment this anti-tumor activity by treatment with a selective RAR agonist. A novel RARγ agonist (IRX4647) was interrogated for in vivo study alone and when combined with an anti-PD-L1 agent given its distinct effects on T cell function^22,23^. Findings were compared to that of an RARα antagonist (IRX6696) because RARα antagonism can repress CD38-mediated immunosuppression^20^.

Intriguingly, syngeneic tumor growth of an immune checkpoint-resistant lung cancer cell line (344SQ) was statistically-significantly reduced after combined IRX4647 and anti-PD-L1 treatments. IRX4647 pharmacokinetics were performed in mice. Cooperative anti-tumorigenic effects were not seen with treatment with an RARα (IRX6696) antagonist. Notably, mice treated with the RARγ agonist IRX4647 and an anti-PD-L1 agent statistically-significantly elevated CD4^+^ T helper cells in the spleen and tumor. This also increased plasma and tumoral IL-5 and IL-13 concentrations. Treating mice with these cytokines altered T cell populations, implicating these changes as eliciting observed anti-tumorigenic effects. Consistent with these results, cultured murine and human lung cancer cells conferred little growth inhibition after IRX4647 treatment. Together, these findings provide a rationale for exploring efficacy of a clinical oncology trial that combines an RARγ agonist with checkpoint blockade.

## Materials and methods

### Cell culture and chemicals

Human lung cancer cell lines (A549, HOP62, and H1299) were purchased and authenticated by American Type Culture Collection (ATCC). Murine lung adenocarcinoma cell lines (344SQ and 393P) were obtained from Dr. Jonathan Kurie at MD Anderson Cancer Center (Houston, TX) and were originally derived from *K-ras*^*LA1/*+^*p53*^*R172HΔg/*+^ engineered mice^24^. The ED1SQ4 murine lung cancer cells were derived in our laboratory from ED1 cells^25^. Murine ED1 lung cancer cells were isolated from cyclin E transgenic mice in our laboratory, as described^25–28^. Cells were cultured in RPMI1640 media (with L-glutamine) supplemented with 10% fetal bovine serum (FBS) in a humidified incubator maintained at 37 °C and with 5% CO_2_. IRX4647 and IRX6696 compounds were obtained from Io Therapeutics, Inc.

### Proliferation assays

Logarithmically growing cancer cells were seeded at optimized densities (8 × 10^2^ cells/well) for each examined cell line onto individual wells of 96-well polystyrene tissue culture-treated microplates (3598, Corning, Corning, NY). Cells were treated with IRX4647 or vehicle (dimethyl sulfoxide, DMSO) at desired concentrations 24 h later. The WST-1 cell proliferation assay (Takara Bio USA, Inc., San Jose, CA) measured cell growth over 5 days using a FLUOstar Omega microplate reader (BMG Labtech, Ortenberg, Germany). These assays were independently repeated at least three times with each biological replicate performed at least in triplicate. Data were normalized to baseline (0 h; no treatment) cell growth.

### Lung cancer murine syngeneic models and drug treatments

All methods are performed and reported in accordance with Animal Research: Reporting of In Vivo Experiments (ARRIVE) guidelines (https://arriveguidelines.org). Animal experiments were conducted following study protocols: #100161 was approved by the National Cancer Institute (NCI) Animal Care and Use Committee (ACUC), and #06-14-04731 was approved by MD Anderson Cancer Center’s Institutional Animal Care and Use Committee (IACUC). Animal euthanasia was with carbon dioxide (CO_2_) inhalation and used the NIH Guideline for Euthanasia of Rodents (https://oacu.oir.nih.gov/system/files/media/file/2021-06/b5_euthanasia_of_rodents_using_carbon_dioxide.pdf). Each mouse was observed for lack of respiration and faded eye color, which was followed by continued CO_2_ flow for a minimum of 1 min after respiration ceased. Murine adenocarcinoma lung cancer 344SQ cells (0.5 × 10^5^ cells) were injected subcutaneously into male 129/Sv mice (Charles River Laboratories, Wilmington, MA). Murine colon adenocarcinoma MC38/gp100 cells (5 × 10^5^) or melanoma BP cells (5 × 10^5^) were injected into C57BL/6 mice (Charles River Laboratories, Wilmington, MA) subcutaneously. ED1SQ4 cells (0.5 × 10^5^ cells) were injected into FVB/N or athymic mice (Charles River Laboratories, Wilmington, MA). The inbred mice were at least 8 weeks old and had at least a one-week acclimation period before experiments began.

Mice with palpable tumors following independent 344SQ or ED1SQ4 lung cancer cell subcutaneous injections were randomized based on similar basal tumor sizes to receive oral doses of vehicle, ATRA, IRX6696 or IRX4647 (1.5 mg/kg body weight; 5 days per week) with or without intraperitoneal injections of PD-L1 antibody (1 mg/ml; twice per week; BP0101; BioXCell, Lebanon, NH). Compound IRX4647 was formulated in 2.5% DMSO/30% PEG400/67.5% Phosal MCT53 solution and treatment results were compared to that of vehicle (2.5% DMSO/30% PEG400/67.5% Phosal MCT53). Body weights and tumor volumes were measured three times weekly. Tumor volumes were calculated as V = (length × width^2^)/2. Tumor, blood, and tissues were harvested at baseline (n = 10; no treatment), after 2 weeks (n = 40), 3 weeks (n = 40), 5 weeks (n = 41), and 8 weeks (n = 48) of treatments.

### Pharmacokinetic studies

Inbred 8–12 weeks old female C57BL/6J mice (The Jackson Laboratory, Bar Harbor, ME) were housed in ventilated cages on a regular 12 h light/dark cycle with ad libitum access to food and water. Animals acclimated for at least one week before a single dose administration of the studied retinoid via oral gavage. Compound IRX4647 was formulated in 2.5% DMSO/30% PEG400/67.5% Phosal MCT53 solution. Plasma and liver from compound-treated mice (n = 5 per time point) were individually harvested at 0.5, 1, 3, 6, 12, 24, and 48 h post-drug administration. Samples were flash frozen and stored at − 80 °C until analysis. Plasma was isolated as were liver homogenates and samples were extracted with acetonitrile and 0.1% formic acid for 30 min at room temperature. Extracts were separated by centrifugation and used for high-performance liquid chromatography (HPLC) analysis. HPLC analyses were performed with a Shimadzu 20AC-XR system. Separation was at room temperature with a Cortecs C18 column (Waters Corp., Milford, MA).

### T cell depletion

Murine lung adenocarcinoma ED1SQ4 (0.5 × 10^5^ cells) or colon adenocarcinoma MC38/gp100 cells (5 × 10^5^ cells) were injected into FVB/N or C57BL/6 mice (Charles River Laboratories, Wilmington, MA) subcutaneously on day 0, respectively. Mice with palpable tumors were randomized based on similar basal tumor sizes to independently receive CD8^+^ T cell depletion antibody (clone HB129/116-13.1, Bioxcell), CD4^+^ T cell depletion antibody (clone GK1.5, Bioxcell, Lebanon, NH), or rat IgG 2b isotype control antibody (Bioxcell) independently on days 5, 7, 13 and 19. Mice were treated with vehicle or ATRA via oral gavage (1.5 mg/kg body weight) on days 7, 8, 9, 12, 13, 14, 15, 16, 19, 20, 21 and 22 before harvesting tumors on day 23. Tumor size was calculated as V = (length × width^2^)/2.

### Flow cytometry

Blood, tissues, and tumors were each freshly harvested and stained with antibodies following the manufacturer’s protocols. Antibodies used were: CD3-PE-Cy7 (560591, BD Biosciences, San Jose, CA), CD4-BV786 (563727, BD Biosciences, San Jose, CA), CD8a-BB515 (564422, BD Biosciences, San Jose, CA), CD19-BB515 (564509, BD Biosciences, San Jose, CA), CD25-BV421 (564370, BD Biosciences, San Jose, CA), CD45-PerCP-Cy5.5 (550994, BD Biosciences, San Jose, CA), CD45RA-PE (553380, BD Biosciences, San Jose, CA), CD49b-BV421 (563063, BD Biosciences, San Jose, CA), CD197-BV650 (564356, BD Biosciences, San Jose, CA), F4/80-BV421 (565411, BD Biosciences, San Jose, CA), CD11b-BV650 (563402, BD Biosciences, San Jose, CA), CD11c-PE (565592, BD Biosciences, San Jose, CA), Ly-6C-BV785 (128041, BioLegend, San Jose, CA), Ly-6G-PE-Cy7 (560601, BD Biosciences, San Jose, CA), RORγt-BV650 (564722, BD Biosciences, San Jose, CA), Foxp3-PE (560408, BD Biosciences, San Jose, CA), or viability stain-510 (564406, BD Biosciences, San Jose, CA). For intracellular staining, cells were fixed and permeabilized with Foxp3/Transcription factor staining buffer set (00-5523-00, ThermoFisher Scientific, Waltham, MA). Data were collected by using a CytoFLEX S flow cytometer (Beckman Coulter Life Sciences, Indianapolis, IN) and analyzed with the CytExpert software (version 2.3; Beckman Coulter Life Sciences, Indianapolis, IN).

### Cytokine analysis

Custom-designed U-Plex multiplex assay kits (Meso Scale Diagnostics, Rockville, MD) independently detected IFN-γ, IL-1β, IL-2, IL-4, IL-5, IL-10, IL-13, IL-17A, IL-21, and TNF-α cytokines in plasma and tumors following the manufacturer’s methods. Data were collected using a MESO QuickPlex SQ 120 instrument (Meso Scale Diagnostics, Rockville, MD) and analyzed with the Discovery Workbench software (version 4.0; Meso Scale Diagnostics, Rockville, MD). Standard curves for each cytokine were generated for calculations of concentrations.

### qRT-PCR assays

Total RNA was isolated using RNeasy Mini Kit (QIAGEN, Hilden, Germany) from cultured cancer cells. The desired cDNA was synthesized from RNA using TaqMan™ reverse transcription reagents (N8080234, ThermoFisher Scientific, Waltham, MA) using the manufacturer’s methods. Real-time qPCR assays were with the TaqMan™ fast advanced master mix (4444963, ThermoFisher Scientific, Waltham, MA) on a QuantStudio™ real-time PCR assay system (ThermoFisher Scientific, Waltham, MA). The mRNA quantification was normalized to β-actin expression. Primers were purchased (ThermoFisher Scientific) for human RARα (Hs00940446_m1), murine RARα (Mm01296312_m1), human RARβ (Hs00233407_m1), murine RARβ (Mm01319677_m1), human RARγ (Hs01559234_m1), murine RARγ (Mm00441091_m1), human β-actin (Hs99999903_m1), and murine β-actin (Mm00607939_s1).

### Immunohistochemistry

Harvested tumors were formalin-fixed and paraffin-embedded. Automated immunohistochemical staining was performed with the Leica Biosystems’ BondRX using Epitope Retrieval 2 (EDTA) for CD38 (ab216343, Abcam, Boston, MA, 1:1000) and CD4 (13-9766, eBioscience, San Diego, CA, 1:250), Epitope Retrieval 1 (Citrate) for CD8a (14-0195-82, eBioscience, San Diego, CA, 1:50) and PD-L1 (AF1019, R&D Systems, Minneapolis, MN, 1:300). The Bond Polymer Refine Detection Kit (DS9800, Leica Biosystems, Wetzlar, Germany) with omission of the PostPrimary Reagent was used. Anti-rat secondary antibody was used for CD4 and CD8; anti-goat secondary antibody was used for PD-L1. Matched isotype antibodies served as negative controls.

Multiplex fluorescent staining was with Leica Biosystems’ BondRX autostainer using the Bond Polymer Refine Kit (DS9800, Leica Biosystems, Wetzlar, Germany), with omission of the PostPrimary reagent, DAB, and Hematoxylin. Antigen retrieval was with Epitope Retrieval 2 (EDTA). Incubations were for 30 min with CD38 (ab216343, Abcam, Boston, MA, 1:1000) followed by the Polymer reagent and OPAL Fluorophore 690 (AKOYA Biosciences, Marlborough, MA). CD38 antibody complex was stripped by heating with Bond Epitope Retrieval 2. Sections were incubated for 30 min with panCytokeratin AE1/AE3 biotin conjugate (NBP2-33200B, NOVUS, Centennial, CO, 1:50), followed by Streptavidin HRP (434323, ThermoFisher Scientific, Waltham, MA) and OPAL Fluorophore 570. Sections were stained with DAPI and coverslipped with Prolong Gold AntiFade Reagent (ThermoFisher Scientific, Waltham, MA). Images were captured using the Aperio Scanscope FL (Leica Biosystems, Buffalo Grove, IL) whole slide scanner.

Image analysis was with Halo imaging analysis software (v3.4.2986.235; Indica Labs, Corrales, NM). Image interpretation was performed by a single reference pathologist. Folds, tears and necrotic regions were excluded from analysis using Densenet AI V2 (Plugin). CD4^+^ T cells, CD8^+^ T cells, and PDL-1 were each quantified using cytonuclear algorithm, v2.0.12. CD38 profiles were quantified using Area quantification FL v2.1.9 in HALO to score percent positive regions of interest.

### Statistical analysis

Data were group means ± standard error of the mean (SEM). Data were analyzed by unpaired Student’s *t*-test and one-way analysis of variance (ANOVA) using GraphPad Prism (version 9.2.0). A *P* value of 0.05 was deemed as statistically-significant.

## Results

### ATRA-treatment repressed syngeneic tumor growth

**Figure 1:**
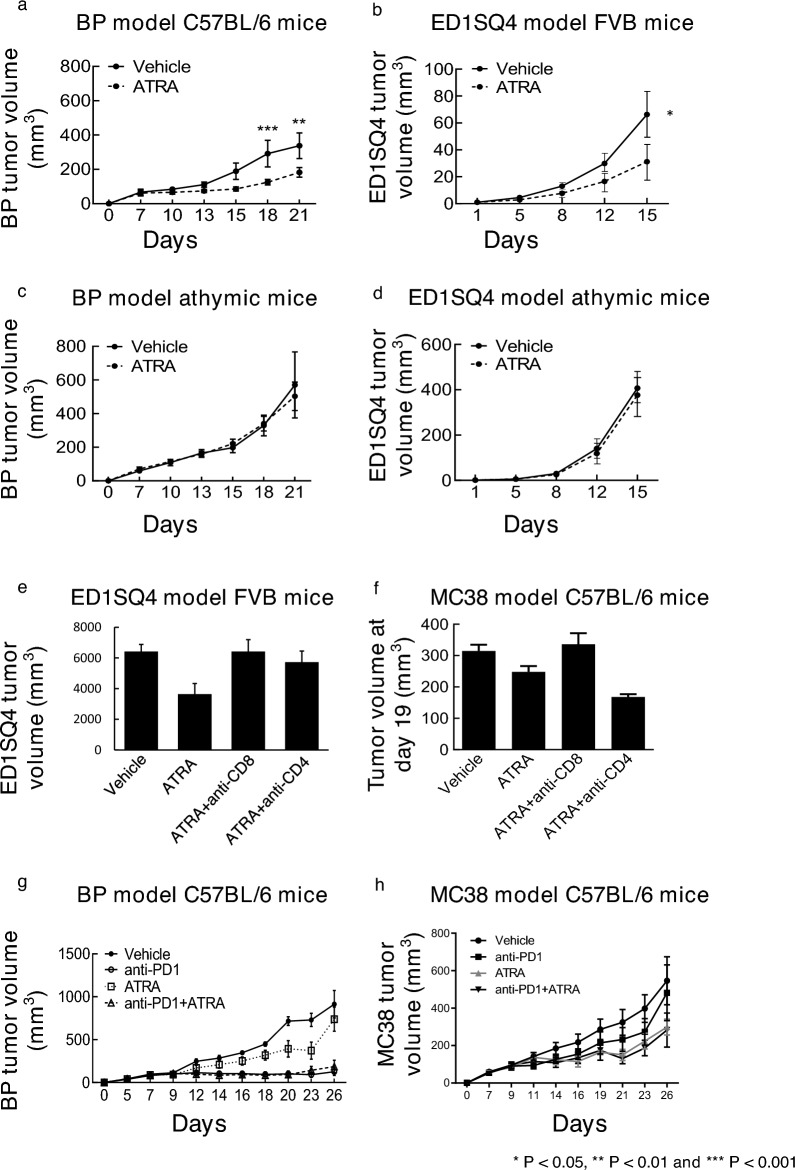
ATRA-treatment repressed syngeneic murine tumor growth. (**a**) Comparison of tumor growth in syngeneic melanoma (BP cells in C57BL/6 mice) treated with ATRA or vehicle. (**b**) Comparison of tumor growth in syngeneic lung cancer (ED1SQ4 cells in FVB mice) treated with ATRA or vehicle. (**c**) Comparison of tumor growth in xenograft melanoma (BP cells in athymic mice) treated with ATRA or vehicle. (**d**) Comparison of tumor growth in xenograft lung cancer (ED1SQ4 cells in athymic mice) treated with ATRA or vehicle. The effect of CD8^+^ or CD4^+^ T cells depletion in syngeneic lung cancer (ED1SQ4 cells in FVB mice) (**e**) and colon cancer (MC38 cells in C57BL/6 mice) (**f**) treated with ATRA. (**g**) Comparison of tumor growth in syngeneic melanoma (BP cells in C57BL/6 mice) treated with vehicle, ATRA, anti-PD1 or combined ATRA+ anti-PD1 treatment. (**h**) Comparison of tumor growth in syngeneic colon cancer (MC38 cells in C57BL/6 mice) treated with vehicle, ATRA, anti-PD1 or combined ATRA+ anti-PD1 treatment. In these displayed experiments, error bars are standard deviations with *P < 0.05, **P < 0.01 and ***P < 0.001, respectively, indicated by the Student’s t test.

Consequences of activation of RARs on the growth of transplanted syngeneic cancers in mice were examined following treatment with the pan-RAR agonist ATRA. Results were compared to that obtained after treatment with vehicle controls. ATRA-treatment independently and statistically-significantly repressed the growth of syngeneic melanoma (BP cells in C57BL/6 mice) and lung cancer (ED1SQ4 cells in FVB mice), as shown in Fig. 1a,b. In marked contrast, ATRA-treatment did not inhibit the growth of xenograft tumors that independently formed after subcutaneous implantation of murine BP or ED1SQ4 cells into immune-deficient mice (Fig. 1c,d). These findings raised the prospect that the reduced tumor growth in mice detected after ATRA-treatment of syngeneic melanoma or lung cancers was exerted through effects on the tumor immune microenvironment.

To investigate whether these observed anti-neoplastic effects were exerted through actions on T cells, cytotoxic (CD8^+^) and helper (CD4^+^) T cells were each depleted. The outcomes on tumorigenicity were assessed in syngeneic C57BL/6 mice bearing transplanted ED1SQ4 lung cancer and MC38 colon cancers, respectively. Notably, CD8^+^ T cell depletion statistically-significantly reduced ED1SQ4 lung cancer and MC38 colon cancer growth inhibition after independent implantation of these cancer cells into syngeneic mice. This implicated ATRA’s tumor-suppressing effects as engaging the actions of T cells, as shown in Fig. 1e,f.

To explore the anti-tumorigenic effects of combining ATRA with an immune checkpoint inhibitor, syngeneic melanoma (BP cells in C57BL/6 mice) and colon cancer (MC38 cells in C57BL/6 mice) were independently treated with vehicle, ATRA, anti-PD1 antibody, or the combined regimen. The combined regimen did not exhibit superior anti-tumorigenic efficacy as compared to the respective monotherapy treatments, as displayed in Fig. 1g,h. These findings raised the possibility that this pan-RAR agonist did not cooperate with immune checkpoint blockage in eliciting anti-tumorigenic effects. Hence, we sought to investigate if an RAR-selective retinoid would have superior anti-neoplastic actions against syngeneic cancers transplanted into mice.

### RAR subtype selective ligands and in vitro cancer cell growth

**Figure 2:**
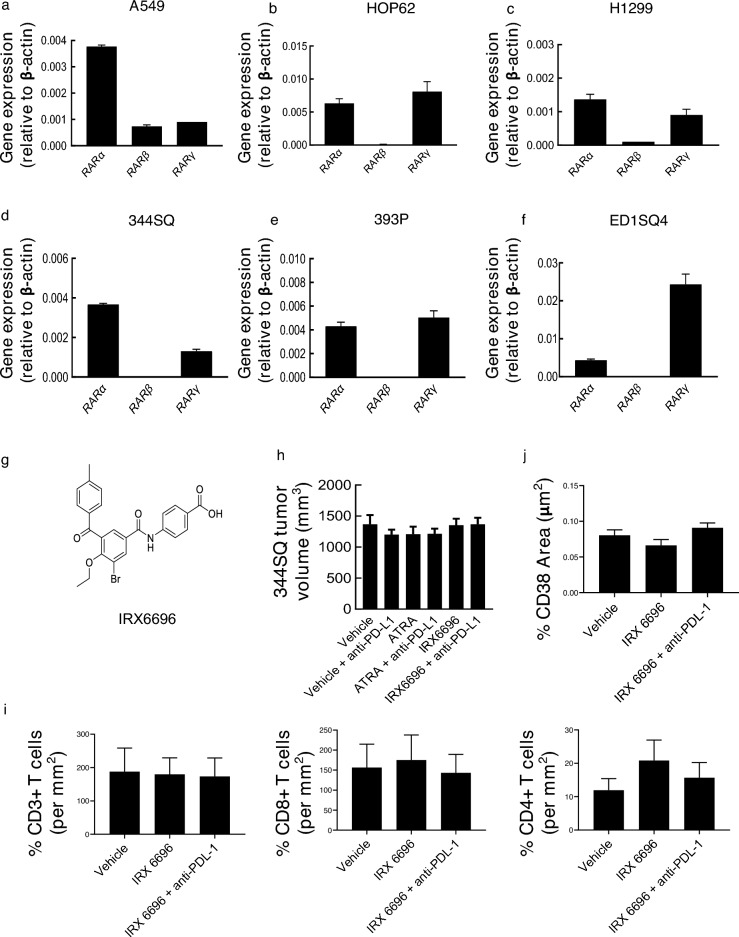
Expression of RARs and effects of RARα antagonist (IRX6696) treatments in lung cancers. Basal expression profiles were independently displayed for RARα, RARβ and RARγ in human (panels **a**–**c**) and murine (panels **d**–**f**) lung cancer cells by real-time PCR assays. The chemical structure of IRX6696 is shown in panel (**g**). (**h**) Comparison of tumor growth in syngeneic lung cancer (344SQ cells in 129sv mice) treated with vehicle, anti-PD-L1, ATRA or in combination with anti-PD-L1, IRX6696 alone or in combination with anti-PD-L1. (**i**) immunohistochemical staining for CD3^+^ , CD8^+^ and CD4^+^ T cells are displayed in lung cancers treated with vehicle, anti-PD-L1, IRX6696 or in combination with anti-PD-L1 and (**j**) Immunofluorescent staining and quantifications of CD38 expression profiles are displayed.

To investigate in vitro actions of targeting cancers with subtype selective RARs, real-time PCR assays were performed to determine the basal expression profiles of RARα, RARβ and RARγ in human and murine lung cancer cells. Results appear in Figs. 2a–f and S1. RARβ mRNA was either undetectable or present at low basal levels while RARα and RARγ mRNAs were each expressed at readily detectable basal levels in these examined cancer cell lines.

Our prior work reported that RARα agonist treatment can augment CD38 expression, leading to acquired lung cancer resistance in mice to anti-PD-L1 therapy^20^. Given this, it was hypothesized that the RARα antagonist IRX6696 would enhance anti-tumorigenic effects of anti-PD-L1 therapy (Fig. 2g). As expected, IRX6696 treatment of cultured human as well as murine lung cancer cells did not appreciably affect proliferation (Fig. S2). Unexpectedly, syngeneic 344SQ lung cancer cells, which are relatively resistant to anti-PDL-1 therapy^20^, and which were subcutaneously injected into 129sv mice before treatment with the RARα antagonist IRX6696 alone, or combined with anti-PD-L1 antibody, did not statistically-significantly affect tumor growth (Fig. 2h).

To elucidate potential mechanisms, immunohistochemcal assays were performed. These assays found that intratumoral T cell (CD3^+^) numbers were not appreciably regulated by IRX6696 treatment and insignificantly increased CD8^+^ and CD4^+^ T cells in the examined tumors (Fig. 2i). Immunofluorescent staining of CD38 of the tumors showed that IRX6696 treatment insignificantly down-regulated CD38 profiles in the fibrovascular stroma and the combination did not down-regulate CD38 in Fig. 2j. Real-time PCR assays found CD38 mRNA levels were not significantly regulated by IRX6696 treatment in tumors (Fig. S3). This finding indicated the need to identify another receptor-selective retinoid that might exert greater anti-neoplastic activity than did IRX6696 against syngeneic tumors in mice.

### IRX4647 pharmacokinetics and in vitro growth effects

**Figure 3:**
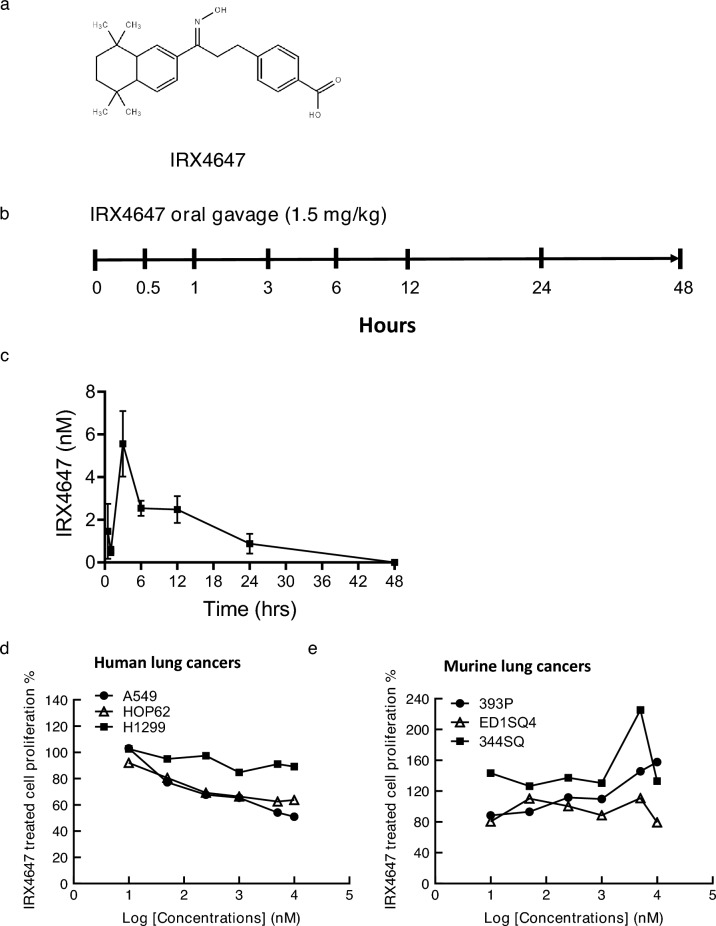
Pharmacokinetics of the RARγ agonist (IRX4647) in mice. (**a**) The chemical structure of IRX4647 is shown. (**b**) IRX4647 was administered to healthy mice through oral gavage. Blood and tissues were harvested at the indicated time points. (**c**) Plasma half-life of IRX4647 was 6 h in mice. IRX4647 did not appreciably affect the proliferation of the examined (**d**) human or (**e**) murine lung cancer cells.

RARγ is known to alter the immune microenvironment^21,22^. It was reasoned that IRX4647, an RARγ selective agonist (Fig. 3a), would augment activity of immune checkpoint inhibitors and overcome resistance to anti-PDL-1 treatments in a syngeneic lung cancer model (344SQ in 129sv mice). An IRX4647 pharmacokinetics study was conducted. IRX4647 was administered to mice as a single dose via oral gavage. Plasma and liver were independently harvested at 0.5, 1, 3, 6, 12, 24, and 48 h post-IRX4647 administration. HPLC and mass spectrometry analyses were performed to identify and quantify IRX4647 levels in study samples. The plasma half-life in mice was determined to be 6 h in Fig. 3b,c. IRX4647 treatment was well-tolerated by mice during this study period. IRX4647-treatment did not appreciably reduce growth of cultured human (A549, HOP62 and H1299) or murine (393P, 344SQ and ED1SQ4) lung cancer cell lines, as displayed in Fig. 3d,e.

### Combined RARγ agonist and anti-PD-L1 treatments

**Figure 4:**
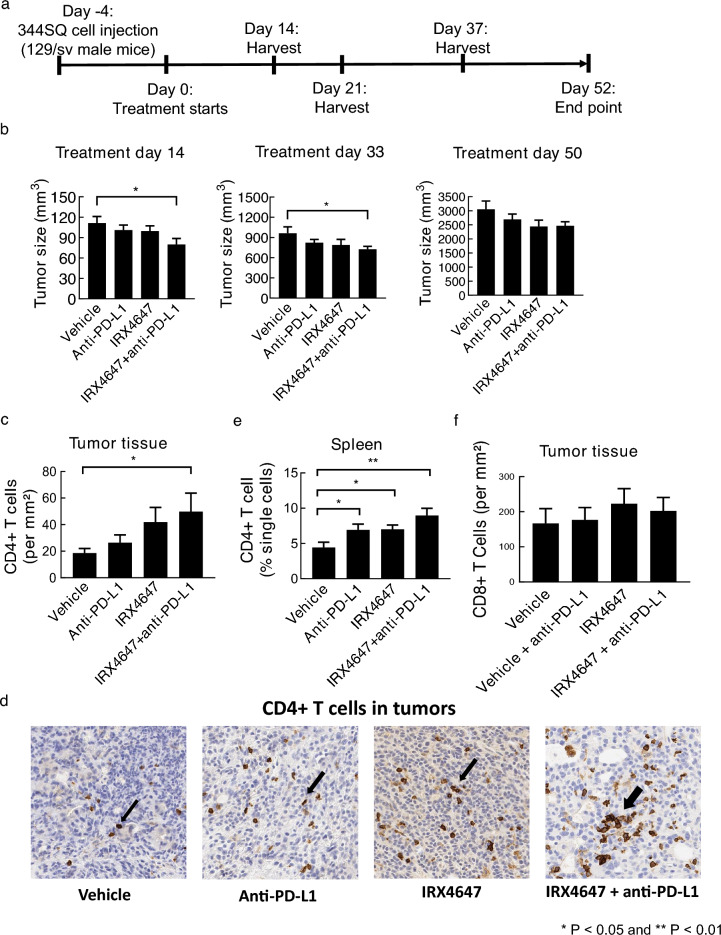
Treatment of mice with the RARγ agonist IRX4647 reduced lung cancer growth in syngeneic mice. (**a**) This diagram displays the treatment regimens used in 344SQ lung cancer-bearing mice treated with vehicle, IRX4647, anti-PD-L1 or the combination. (**b**) The combination regimen treatment statistically-significantly reduced syngeneic lung cancer growth as measured on day 14 and 33. (**c**) Immunohistochemical staining showed that this regimen augmented intratumoral CD4^+^ T cells, with representative images shown in panel (**d**). (**e**) Flow cytometry assays showed increased CD4^+^ T cells present in the spleen. (**f**) Immunohistochemical staining showed that IRX4647-treatment did not statistically-significantly increase intratumoral CD8^+^ T cells.

To elucidate whether combining an RARγ agonist with checkpoint blockage can overcome the relative anti-PDL-1 resistance of murine 344SQ lung cancer cells^20^ that were implanted subcutaneously into syngeneic mice, palpable tumors were treated with IRX4647 as a single agent or in combination with anti-PD-L1 antibody, as in Fig. 4a. The combined IRX4647 and anti-PD-L1 antibody regimen statistically-significantly reduced syngeneic lung cancer growth. Anti-neoplastic effects were evident at as early as day 14 after treatments began and continued through day 33. These treatments exhibited tumor growth suppression even at day 50, as in Fig. 4b.

To investigate underlying mechanisms, immunohistochemical assays were conducted on harvested tumors. Immunohistochemical expression profiles found that tumor-infiltrating CD4^+^ T cells statistically-significantly increased after treatment of these lung cancers with the combined regimen as compared to vehicle-treatments (Fig. 4c,d). Flow cytometry assays were performed and established that the CD4^+^ T cell population was statistically-significantly augmented within splenic tissues after individual anti-PD-L1 or IRX4647 treatments and an even more prominent increase occurred after treatment with this combined regimen (Fig. 4e). In Fig. 4f immunohistochemical staining revealed that IRX4647-treatment did not statistically-significantly increase intratumoral CD8^+^ T cells.

### IRX4647 treatment reduces CD38 expression in the stroma

**Figure 5:**
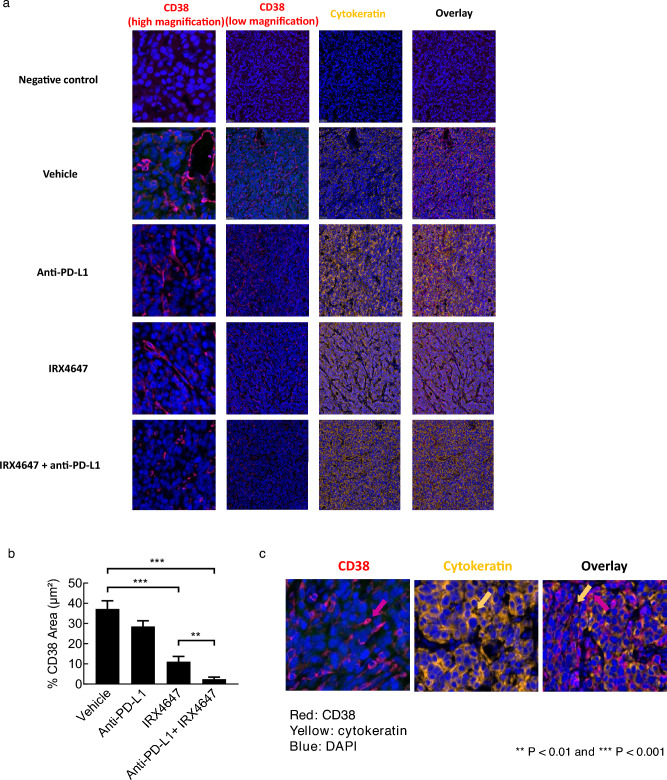
Immunofluorescent staining of CD38 (red) and cytokeratin (yellow) revealed that CD38 expression was predominately detected in the fibrovascular stroma and was statistically-significantly reduced in the IRX4647 and anti-PD-L1 treatment groups. Even greater repression occurred in the combined treatment group. The representative images are displayed in panel (**a**); quantification appears in panel (**b**). (**c**) Higher magnifications of representative images are presented that depict CD38 (red) and cytokeratin (yellow) signals in the different treatment groups.

Immunofluorescent staining was next performed to investigate whether CD38 expression levels were regulated by IRX4647 or anti-PD-L1 treatment in subcutaneous syngeneic 344SQ lung cancer cells present in mice treated with vehicle, IRX4647, anti-PD-L1, or the combined regimen, as shown in Fig. 5a. Notably, CD38 immunohistochemical expression profiles in the fibrovascular stroma were statistically-significantly reduced in the IRX4647 or anti-PD-L1 treatment groups. An even greater statistically-significant repression was detected after combination regimen treatments, as in Fig. 5b. The CD38 expression profiles in these tumors were typically undetected as compared to that evident in fibrovascular stroma. A higher magnification of the immunohistochemical profiles of CD38 as compared to cytokeratin expression appears in Fig. 5c. These data confirm the fibrovascular stromal expression pattern for CD38. These results imply that the combined antineoplastic effects in these syngeneic tumors after individual or combined IRX4647 and anti-PD-L1 treatments were not mediated only by reduced CD38 expression within these syngeneic lung cancers. Thus, we sought to identify candidate mediators of this antitumorigenic activity.

### IL5 and IL13 increase CD4^+^ and CD8^+^ T cell populations

**Figure 6:**
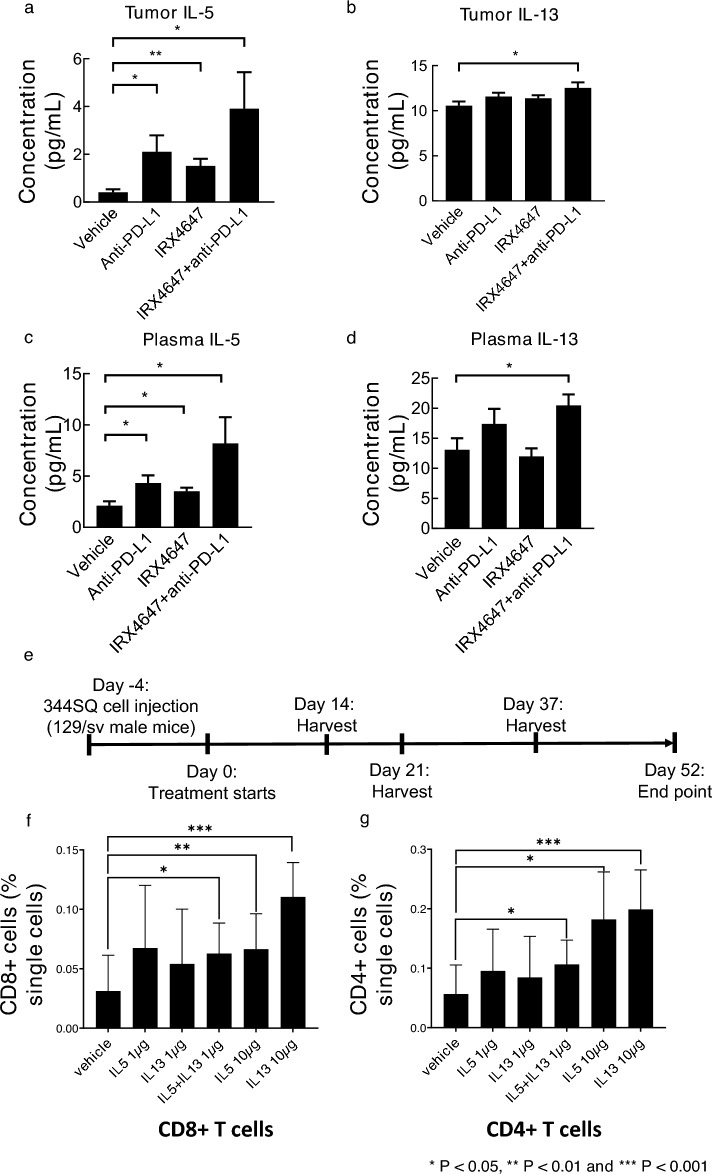
IRX4647-treatment increased both IL-and IL13 expression in syngeneic lung cancers. Intratumoral (**a**) IL-5 and (**b**) IL-13 levels, as well as levels for these cytokines in plasma are displayed in panels (**c**) for IL-5 and (**d**) for IL-13. These levels were statistically-significantly increased in tumors treated with IRX4647, anti-PD-L1 or the combined regimen. Lung cancers for these cytokine analyses were harvested at the time points shown in panel (**e**). Treatment with IL-5, IL-13, or the combined therapy in 129sv mice increased CD8^+^ T cells populations and CD4^+^ T cells expression profiles as shown in panels (**f**) and (**g**), respectively.

Cytokine analysis of lysates isolated from these lung tumors established that IL-5 and IL-13 levels were statistically-significantly increased in tumors treated with IRX4647, anti-PD-L1 or these combinations (Fig. 6a,b) as well as in the plasma from the same mice as shown in Fig. 6c–e. These other cytokines were also examined: IL-2, IL-17A, IL-4 or IL-21. These cytokines were augmented more often after anti-PD-L1 than after IRX4647 treatments (Fig. S4). Prior work reported that IL-5 and IL-13 cytokines can activate CD4^+^ T cells^29,30^. We sought to examine whether treatment of mice with IL-5 alone, IL-13 alone or both cytokines together affected different T cell populations. IL-5, IL-13 or both cytokines were administered to wild-type 129/sv mice. Blood was harvested from mice to measure different T cell populations. Interestingly, CD4^+^ and CD8^+^ T cells were statistically-significantly increased after individual or combined treatments with IL-15 and IL13, as displayed in Fig. 6f,g.

## Discussion

Augmenting clinical efficacy of immune checkpoint blockade is currently an unmet medical need. Exploring combination therapies that engage clinically-tractable mechanisms warrant study. ATRA is the vitamin A metabolite that regulates diverse signaling pathways including those that engage pro-tumoral and anti-tumoral immune effects^1,5,8^. There is a knowledge gap that currently exists as to how the RAR subtype-selective retinoids like the RARγ agonist IRX4647 or the RARα antagonist IRX6696 affect cancer immunity. There is yet another major gap in our understanding of retinoid effects on immunity. This is how RAR-selective agents affect checkpoint blockade response. This study sought to address these substantial gaps in our understanding of retinoid pharmacology.

The findings presented here indicate that ATRA-treatment repressed growth of transplanted syngeneic murine cancer models. Yet, this therapeutic outcome was lost in immunodeficient mice (Fig. 1a–d). Combining ATRA-treatment with immune checkpoint blockade did not cooperatively inhibit lung cancer growth in syngeneic mice (Fig. 1e–h). Both RARα and RARγ were expressed in the examined human and murine lung cancer cell lines (Fig. 2a–f). Given this and our prior work^17–20^, it was hypothesized that an RARα antagonist (IRX6696) would cooperate with anti-PD-L1 therapy to elicit an anti-tumor response to a previously resistant syngeneic lung cancer by reducing CD38 expression and thereby conferring anti-neoplastic effects. However, this treatment regimen did not exhibit the desired anti-tumor actions (Fig. 2g–j). This indicated that another RAR-selective agent and a mechanism other than reduced CD38 expression alone were needed to augment therapeutic consequences of checkpoint blockade.

The RARγ agonist, IRX4647, had superior anti-tumorigenic effects as compared to the RARα antagonist, IRX6696, alone or when added to checkpoint blockade. Combining IRX4647 with checkpoint blockade (anti-PD-L1) reduced in vivo growth of the murine syngeneic 344SQ lung cancer model (Fig. 4a,b). Notably, IRX4647-treatment increased intratumoral as well as splenic CD4^+^ T cells (Fig. 4c–e). IRX4647 also augmented levels of IL-5 and IL-13 cytokines in examined murine tumors and plasma (Fig. 6a–e). Individual or combined IL-5 and IL-13 cytokine treatments increased CD4^+^ and CD8^+^ T cells in mice (Fig. 6f–g). This finding links changes in expression of these cytokines to the observed anti-neoplastic effects. The differential effects of IL-5 and IL-13 on CD8^+^ or CD4^+^ T cells were likely due to a dose-dependent effect, where more CD4^+^ T cells were produced than were CD8^+^ T cells (Fig. 6f–g). Notably, CD38 expression decreased in the tumor fibrovascular stroma after IRX4647-treatment (Fig. 5).

These findings indicate that pharmacologically-mediated reduction of CD38 levels in the tumor microenvironment alone did not elicit anti-tumorigenic responses in syngeneic lung cancers that are basally resistant to checkpoint blockade. Consistent with this pre-clinical finding was clinical evidence from two independent Phase I/II open label clinical trials in patients with advanced solid tumors, including lung cancer cases^31,32^. Those trials combined respectively the anti-CD38 antibody isatuximab with an anti-PD-L1 antibody (atezolizumab) in immune naive solid tumors or following targeting CD38 with isatuximab and PD-1 with cemiplimab in immune naive patients with metastatic castration-resistant prostate cancer cases or in non-small cell lung cancer (NSCLC) cases that progressed on anti-PD-1/PD-L1-combined therapy^31,32^. While both regimens were well tolerated neither trial exerted substantial anti-tumorigenic activity^31,32^.

What appeared to exert anti-tumorigenic effects in this current work was the increased presence of intratumoral CD4^+^ T cells. This was observed following treatment of mice with the RARγ agonist IRX4647, but not with the RARα antagonist, IRX6696. CD4^+^ T cells are components of the immune system that play a major role in conferring immune checkpoint response^33^. Prior work found that anti-PD-L1 therapy enhances the activity of CD4^+^ T cells, leading to increased tumor infiltration and improved anti-tumor activity^34^. Notably, CD4^+^ T cell presence in the tumor microenvironment is one of the key mechanisms that underly the observed effects of IRX4647-treatment on immune response and on anti-neoplastic effects. The combined regimen of IRX4647 and anti-PD-L1 treatments cooperated to promote the appearance of infiltrating CD4^+^ T cells in the syngeneic tumor, as shown in Fig. 4c,d.

It is notable that this study used a regimen that combined an RARγ agonist with checkpoint blockade to repress lung cancer growth. This syngeneic murine lung cancer model was relatively resistant to checkpoint blockade^20^. This raises the prospect that this regimen will augment the activity of checkpoint blockade beyond that observed in pre-clinical murine cancer models. It remains to be discerned if these promising pre-clinical findings will translate into the cancer clinic. Pertinent to the findings presented here is that a clinical trial is underway exploring the efficacy of combining anti-PD-L1 agent (Avelumab) with recombinant IL-5 for subtypes of lymphoma (NCT03905135). Based on the findings provided here, it is expected that recombinant IL-5 would increase intratumoral CD4^+^ T cell populations. It is also hypothesized that use of an RARγ agonist would further augment anti-neoplastic actions of this cytokine.

Before any clinical trial is conducted with the RARγ agonist IRX4647 pre-clinical toxicology is indicated. This is needed to limit the potential clinical toxicity of this retinoid in patients. Arguing for conduct of these studies is that in mice IRX4647 has favorable pharmacokinetics, as shown in Fig. 3. Comprehensive IRX4647 pharmacokinetic studies are warranted in human subjects. These analyses will help determine the optimal pharmacokinetics that can be exploited in the clinic. Further rationale for conducting a clinical trial with this or another RARγ agonist comes from the lack of ability of ATRA or the RARα antagonist to augment anti-tumorigenic activity with anti-PD-L1 blockade, as displayed in Fig. 2h.

One approach to consider in clinical development of IRX4647 is to perform a proof of principle clinical trial with this RARγ agonist. We successfully used this approach in prior clinical trials to learn if a drug of interest reaches its target and elicits desired pharmacodynamic effects^35–38^. For analysis of the anti-tumorigenic effects of IRX4647 in a proof of principle lung cancer trial, it would prove informative to determine pharmacokinetic and pharmacodynamic actions in pre-treatment versus post-treatment tumor biopsies. Findings could then be compared to those obtained after combined IRX4647 and checkpoint blockade treatments. These studies would determine if either treatment promotes the presence of intratumoral CD4^+^ T cells and thereby exerts anti-tumorigenic effects in lung or other cancer types. Positive findings from proof of principle trials would provide a basis for subsequent Phase I or Phase II clinical trials. A similar strategy could analyze the anti-tumorigenic activity of individual IL-5 or IL-13 cytokines or following a combined IL-5 and IL-13 regimen with checkpoint blockade.

Taken together, the findings reported here provide a strong rationale to explore efficacy of combining an RARγ agonist with immune checkpoint blockade in lung cancer therapy. Similar ATRA-mediated anti-neoplastic effects were observed in syngeneic murine cancers in addition to those found in lung cancers. These include colon and melanoma, as shown in Fig. 1. But unlike combined checkpoint blockade treatment with the RARγ agonist IRX4647, such a regimen with ATRA did not statistically-significantly reduce syngeneic cancer growth in mice, as displayed in Fig. 1. We hypothesize that an RARγ agonist like IRX4647 could exert substantial anti-neoplastic actions as a single agent or when combined with checkpoint blockade to augment anti-tumor responses in diverse human cancers. Future clinical trials should explore these intriguing possibilities.

### Supplementary Information

Supplementary Figures.

## Data Availability

The datasets used and/or analyzed during the current study are available from the corresponding author upon reasonable request.
