# Supplementary material for: A novel retinoic acid receptor-γ agonist antagonizes immune checkpoint resistance in lung cancers by altering the tumor immune microenvironment
Source: Sci Rep. 2023 Sep 9;13:14907. doi: 10.1038/s41598-023-41690-5 (PMC10492813; doi:10.1038/s41598-023-41690-5)
Supplement: Supplementary file 1 — Supplementary Figures. [file 41598_2023_41690_MOESM1_ESM.pptx]

## Slide 1
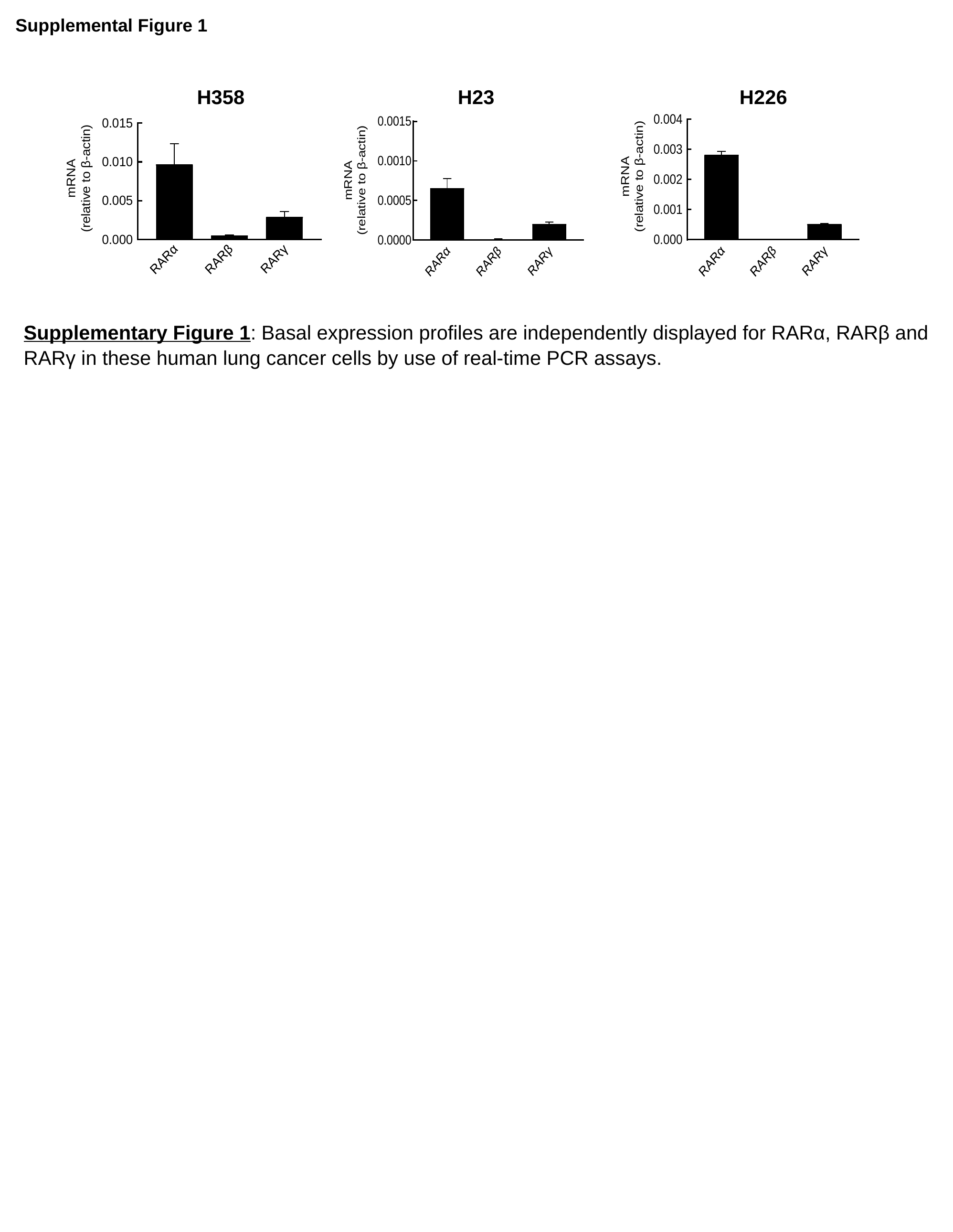

Supplemental Figure 1
H358
H23
H226
Supplementary Figure 1: Basal expression profiles are independently displayed for RARα, RARβ and RARγ in these human lung cancer cells by use of real-time PCR assays.

## Slide 2
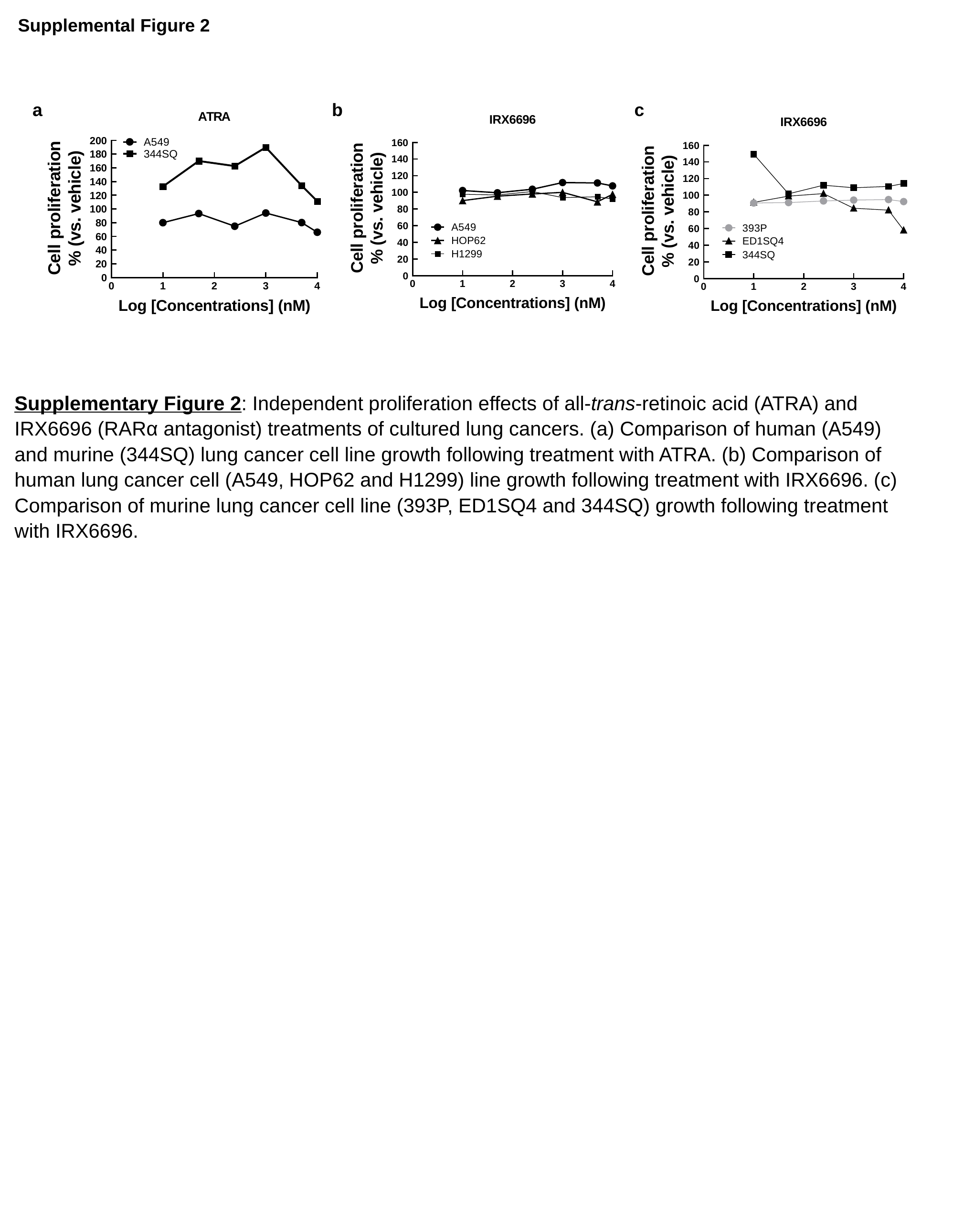

Supplemental Figure 2
a
b
c
Supplementary Figure 2: Independent proliferation effects of all-trans-retinoic acid (ATRA) and IRX6696 (RARα antagonist) treatments of cultured lung cancers. (a) Comparison of human (A549) and murine (344SQ) lung cancer cell line growth following treatment with ATRA. (b) Comparison of human lung cancer cell (A549, HOP62 and H1299) line growth following treatment with IRX6696. (c) Comparison of murine lung cancer cell line (393P, ED1SQ4 and 344SQ) growth following treatment with IRX6696.

## Slide 3
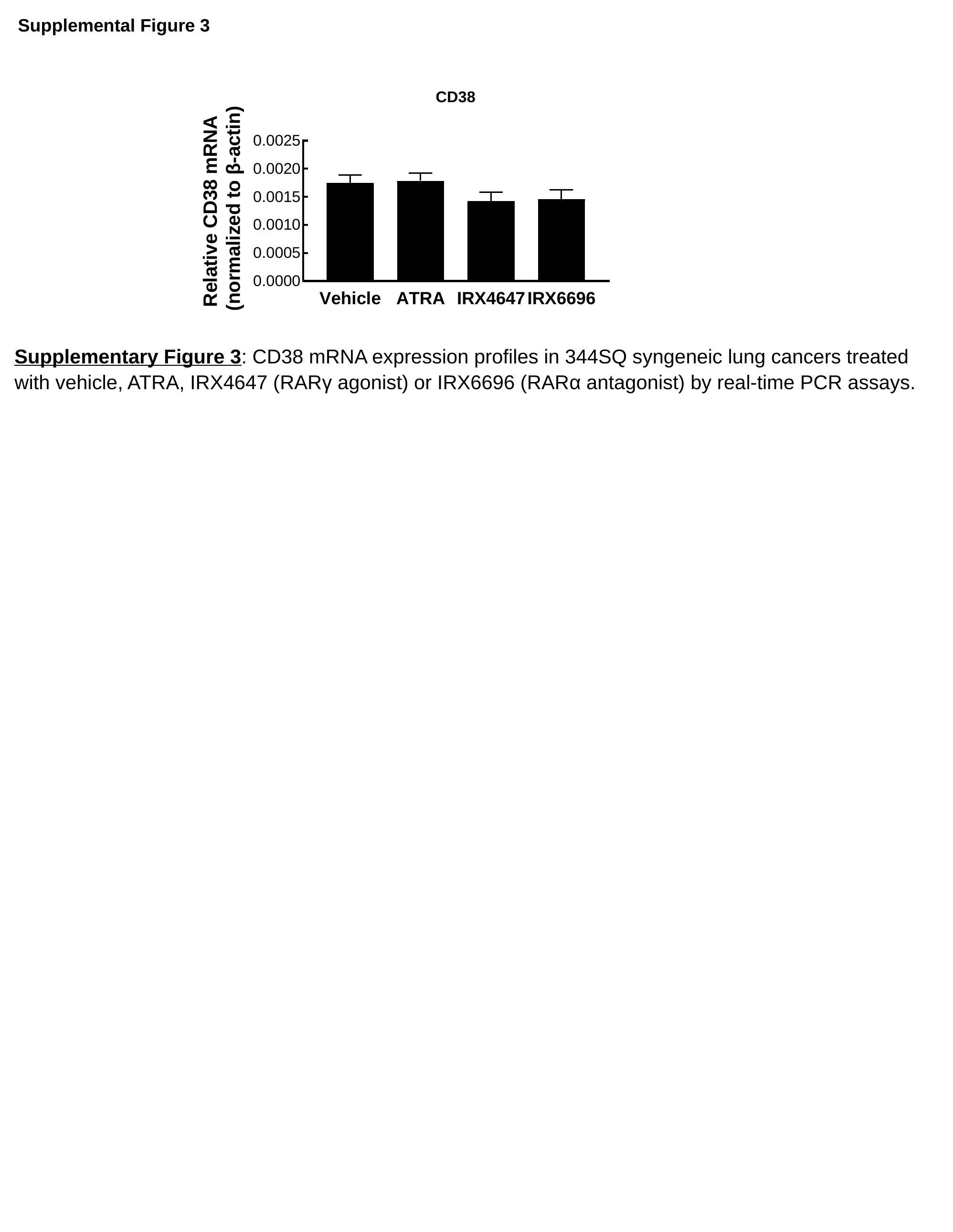

Supplemental Figure 3
Supplementary Figure 3: CD38 mRNA expression profiles in 344SQ syngeneic lung cancers treated with vehicle, ATRA, IRX4647 (RARγ agonist) or IRX6696 (RARα antagonist) by real-time PCR assays.

## Slide 4
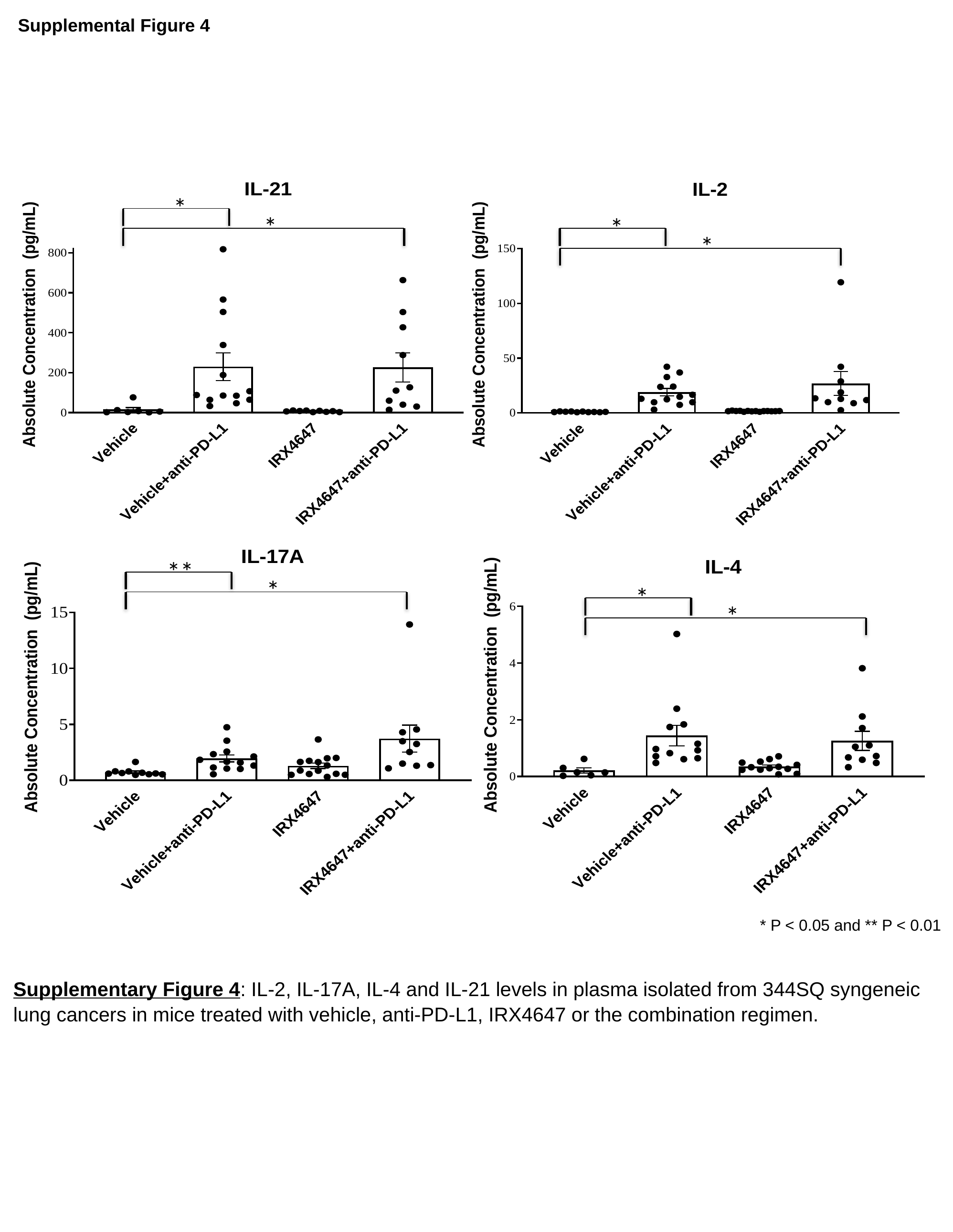

Supplemental Figure 4
*
*
*
*
**
*
*
*
* P < 0.05 and ** P < 0.01
Supplementary Figure 4: IL-2, IL-17A, IL-4 and IL-21 levels in plasma isolated from 344SQ syngeneic lung cancers in mice treated with vehicle, anti-PD-L1, IRX4647 or the combination regimen.
